# A Paradoxical Triad: Scapulothoracic Dissociation with Clavicle and Humeral Shaft Fractures

**DOI:** 10.1155/2014/689157

**Published:** 2014-07-22

**Authors:** Sandeep Albert, Viswanath Jayashankar, Mohamad Gouse

**Affiliations:** Department of Orthopaedics, Christian Medical College, Unit 1, Vellore 632004, India

## Abstract

Scapulothoracic dissociation involves varying degree of discontinuity of the upper extremity from its truncal attachment. An eighteen-year-old male presented to the accident and emergency department following a motor vehicle accident where he was hit by a four wheeler while riding a two wheeler. He had tenderness and deformity over the left clavicle and the left humerus. He was unable to perform active wrist and finger dorsiflexion. A CT subsequently revealed a grade 2 splenic laceration. The splenic laceration was treated conservatively. As his general condition improved, he was gradually weaned off the ventilator and his left upper limb neurology was reassessed. He had isolated radial nerve palsy with an otherwise intact brachial plexus. He underwent internal fixation of the clavicle and the humerus. At 4 months after injury the EMG/NCV report showed signs of renervation of the radial nerve, and the fracture progressed to an uneventful union. This prior unreported triad of scapulothoracic dissociation with ipsilateral clavicular and humeral fractures may represent a parody. An apparent increase in the severity of skeletal injury was associated with a paradoxical decrease in the severity of neurovascular injury. We report this case to create awareness among orthopedic surgeons and emergency physicians about the clinical presentation of such injuries.

## 1. Introduction

As the name suggests scapulothoracic dissociation involves varying degree of discontinuity of the upper extremity from its truncal attachment. It has also been described as “a closed forequarter amputation” [1]. The zone of injury involves musculature and neurovascular structures and is associated with significant morbidity [2]. The injury is either due to a strong traction force or a direct blunt injury. We describe a patient who presented with scapulothoracic dissociation with ipsilateral fractures of the clavicle and humeral shafts which together constitute a triad of injuries which may have had an impact on its severity.

## 2. Case Report

**Figure 1:**
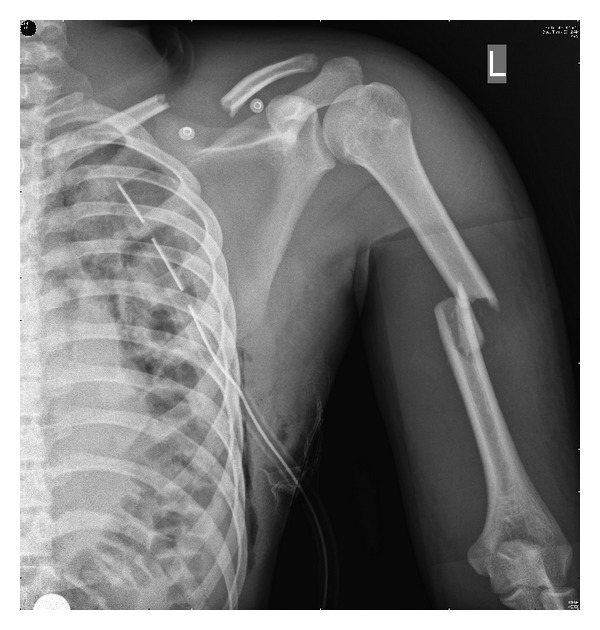
Plain radiograph showing a displaced fracture of clavicle and comminuted fracture shaft of humerus.

**Figure 2:**
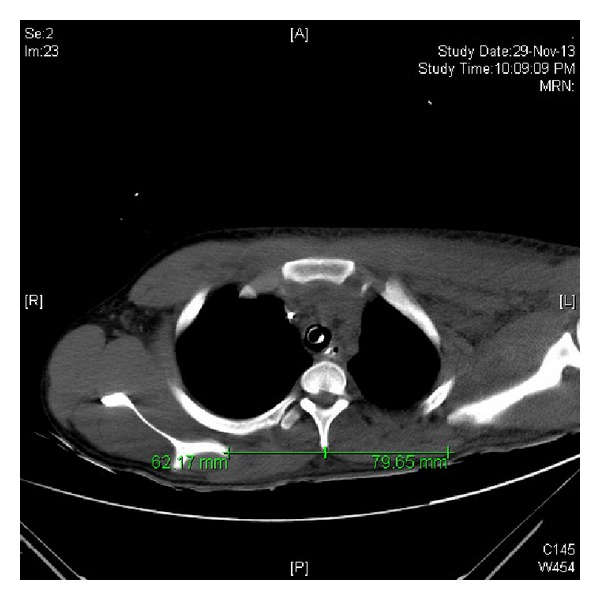
Axial CT section revealing the increase in distance between medial border of left scapula and the spinous process of the fourth thoracic vertebra.

**Figure 3:**
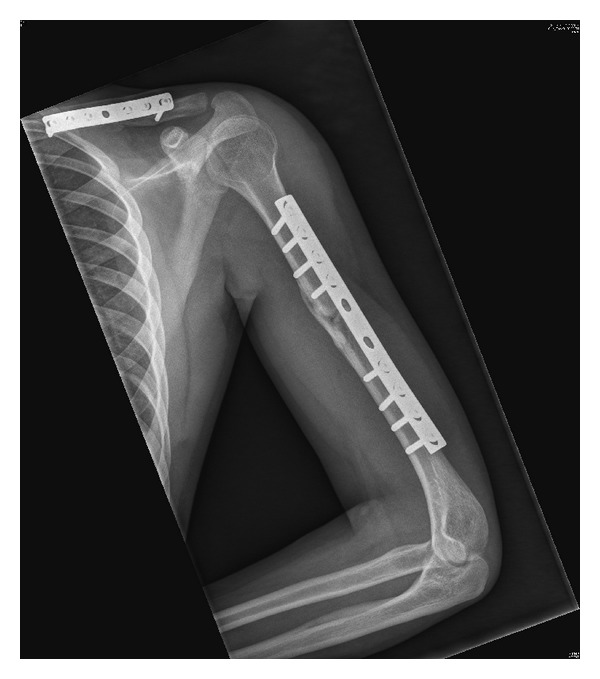
Follow-up radiograph showing union of the clavicle and humerus.

An eighteen-year-old male presented to the accident and emergency department following a motor vehicle accident where he was hit by a four wheeler while riding a two wheeler. On examination he was conscious with a GCS of 15/15 tachycardic, hypotensive, and tachypnoeic. He had diffuse fullness over the left shoulder and scapula and decreased breath sounds over the left hemithorax. He had tenderness and deformity over the left clavicle and the left humerus. He was unable to perform active wrist and finger dorsiflexion. Examination of the abdomen revealed tenderness and guarding over the left upper quadrant. His distal pulses in all four limbs were palpable and symmetrical. An ultrasound abdomen revealed presence of free fluid in the abdomen. A CT subsequently revealed a grade 2 splenic laceration. The initial chest radiograph in accident and emergency (Figure 1) showed a displaced left clavicle fracture and widening between the left medial border of the scapula and the thoracic spine (Figure 2). He also had a comminuted shaft of humerus fracture. In view of persisting hypotension and shallow respiration he had emergency intubation and later shifted to surgical intensive care. The splenic laceration was treated conservatively and he was appropriately transfused. As his general condition improved, he was gradually weaned off the ventilator and his left upper limb neurology was reassessed. He had an isolated radial nerve palsy with an otherwise intact brachial plexus. The patient was counseled about the nature of the injury and he subsequently underwent internal fixation of the clavicle and humerus. Physiotherapy and mobilization were instituted to prevent contractures and his neurological status was monitored. At 4 months after injury the EMG/NCV report showed signs of renervation of the radial nerve, and the fracture progressed to an uneventful union (Figure 3).

## 3. Discussion

Scapulothoracic dissociations represent a spectrum of morbid upper limb injuries around the shoulder for which one of the most important prognostic factors is the extent of neurological injury [3]. Such severe traction or direct impact mechanism is likely to have a wide zone of injury and has been associated with osseous injuries around the shoulder girdle. Masmejeam et al. reported a 10% mortality and nearly 88%–100% risk of associated vascular injuries [4]. The prevalence of brachial plexus injuries was also high (94%) and usually complete [5]. Lange and Noel postulated that the mechanism of the injury resulting in proximal avulsion of the brachial plexus was due to intact clavicle acting as a fulcrum [6]. In our patient the fulcrum, that is, clavicle, was fractured and this may be the reason for the absence of major neurovascular injury. The radial nerve deficit, in this case, could be attributed to the humeral shaft fracture. To our knowledge scapulothoracic dissociation with a combination involving ipsilateral clavicular and humeral shaft fracture has not been described. The peculiar attribute in this patient is the absence of a major vascular or a dense brachial plexus lesion.

Earlier described as case reports or as case series, these injuries were characterized by near universal presence of a neurovascular injury. Several centers have noted that despite early resuscitation and reconstruction patients rarely went on to have a functional limb. Damschen et al. has even advocated an early amputation in a flail limb [5]. The patient described in this case report sustained his injuries following a collision with a four wheeler while riding a two wheeler. The effect of a direct impact injury on the shoulder and brachial plexus may have been reduced in the presence of a fractured clavicle and humerus. This may explain the absence of severe vascular or a dense neurological deficit. An et al. noted that, in children, these morbid injuries presented with an insignificant neurovascular injury probably due to the elasticity of soft tissue structures [7].

## 4. Conclusion

This prior unreported triad of scapulothoracic dissociation with ipsilateral clavicular and humeral fractures may represent a parody. An apparent increase in the severity of skeletal injury was associated with a paradoxical decrease in the severity of neurovascular injury. Closer scrutiny of such injury patterns in large trauma centres, data bases, and prior case series may be required to further validate our conclusion. We report this case to create awareness among orthopedic surgeons and emergency physicians about the clinical presentation of such injuries.
